# Controls on soil microbial community stability under climate change

**DOI:** 10.3389/fmicb.2013.00265

**Published:** 2013-09-05

**Authors:** Franciska T. de Vries, Ashley Shade

**Affiliations:** ^1^Faculty of Life Sciences, The University of ManchesterManchester, UK; ^2^Department of Molecular, Cellular, and Developmental Biology, Yale UniversityNew Haven, CT, USA

**Keywords:** disturbance, drought, fungi, bacteria, PLFA, pyrosequencing, resistance, resilience

## Abstract

Soil microbial communities are intricately linked to ecosystem functioning because they play important roles in carbon and nitrogen cycling. Still, we know little about how soil microbial communities will be affected by disturbances expected with climate change. This is a significant gap in understanding, as the stability of microbial communities, defined as a community's ability to resist and recover from disturbances, likely has consequences for ecosystem function. Here, we propose a framework for predicting a community's response to climate change, based on specific functional traits present in the community, the relative dominance of r- and K-strategists, and the soil environment. We hypothesize that the relative abundance of r- and K-strategists will inform about a community's resistance and resilience to climate change associated disturbances. We also propose that other factors specific to soils, such as moisture content and the presence of plants, may enhance a community's resilience. For example, recent evidence suggests microbial grazers, resource availability, and plant roots each impact on microbial community stability. We explore these hypotheses by offering three vignettes of published data that we re-analyzed. Our results show that community measures of the relative abundance of r- and K-strategists, as well as environmental properties like resource availability and the abundance and diversity of higher trophic levels, can contribute to explaining the response of microbial community composition to climate change-related disturbances. However, further investigation and experimental validation is necessary to directly test these hypotheses across a wide range of soil ecosystems.

## Introduction

Soil microbial communities are intricately linked to ecosystem functioning because they play important roles in carbon (C) and nitrogen (N) cycling, and feed back to plant communities as mutualists and pathogens (Van der Heijden et al., 2008). Although much research has been done to study the impacts of a range of disturbances on soil microbial communities and their functioning (Griffiths and Philippot, 2013), many uncertainties remain about the controls on soil microbial community stability (Box 1), and the consequences of disturbance-induced changes in microbial communities for their capacity to withstand further disturbances. This may be in part because most studies measured the stability of bulk microbial properties, such as biomass and respiration, rather than of community structure (the number of different taxa and their relative abundances; Box 1). However, changes in the abundances or relative contributions of community members may have implications for the stability of a microbial community, and these kinds of membership changes may not be apparent when measuring bulk microbial properties. In addition, soils are unique and highly heterogeneous environments, and controls on microbial community stability in soil might differ from other systems. We argue that knowledge on what controls soil microbial community stability is pivotal for predicting the impacts of climate change on soil microbial communities and the processes that they drive.

Box 1 **Glossary**

*Microbial community composition*: the assortment of microbial taxa that comprises a community (Hunter, 1990).

*Microbial community structure*: the membership and (relative) abundances of microbial taxa in a community (Anderson et al., 2011).

*Trait*: phenotypic characteristic or attribute of an individual microbe that is affected by genotype and the environment (Campbell and Reece, 2006).

*Functional trait*: trait with a direct functional role that defines a microbe in terms of its ecological role, i.e., its interaction with other microbes and its environment (Lavorel and Garnier, 2002; Wallenstein and Hall, 2012).

*Disturbance*: causal event that alters a community directly or indirectly, typically through its effect on the community's environment (Rykiel, 1985; Glasby and Underwood, 1996).

*Pulse disturbance*: relatively discrete (with a clear start and end point), short-term events with a clear start and end point (Lake, 2000).

*Press disturbance*: long term event or continuous change (Lake, 2000).

*Climate change*: statistically significant variation in the mean state of the climate or its variability, caused either by natural internal processes or external forcing, or by persistent anthropogenic-induced changes in the composition of the atmosphere or land use (IPCC, 2007). Here, we focus on disturbances associated with climate change that are relevant to soil communities and processes, namely elevated atmospheric CO_2_ and its indirect effects (increased soil C inputs through roots, root exudates, and increased litter fall), extreme weather events (drought and heavy rainfall), and warming.

*Global change*: changes in the global environment that may alter the capacity of the Earth to sustain life (Schlesinger, 2006), including both land-use and climate change. Here, we focus on global change disturbances such as land use change and N deposition rather than on climate change disturbances.

*Stability*: the tendency of a community to return to a mean condition after a disturbance (Pimm, 1984); includes the components of resistance and resilience (see also Worm and Duffy, 2003; Shade et al., 2012a).

*Resistance*: the ability of a community property or process to remain unchanged in the face of a specific disturbance (Pimm, 1984; Allison and Martiny, 2008).

*Resilience*: the ability of a community property or process to recover after a specific disturbance, often reported as a rate of return (Allison and Martiny, 2008).

*Adaptation*: the process through which a microbe increases its fitness in a particular environment (Wallenstein and Hall, 2012), i.e., optimization of traits that increase fitness.

*Evolutionary adaptation*: changes in the relative abundance of gene frequencies in a gene pool to optimize traits that increase fitness as a result of changes in environmental conditions (Campbell and Reece, 2006; Orsini et al., 2013).

Here, drawing from findings from both terrestrial and aquatic systems, we formulate hypotheses on the controls of resistance and resilience of microbial communities in soil, focusing on disturbances associated with climate change (Box 1). Climate change is expected to result in increased frequency of drought and heavy rainfall, increases in temperature, and increased litter inputs and plant root exudates through elevated concentrations of atmospheric CO_2_, which all have significant impacts on soil microbial community structure and functioning (Bardgett et al., 2013). Here, we focus on pulse disturbances associated with climate change, such as drought, increased rainfall, and increased litter inputs, because the clear start and end point of these disturbances allows for assessing both resistance and resilience of microbial community composition (Box 1). We use three case studies in which we re-analyze published data on the impact of these disturbances on microbial communities to further develop our proposed hypotheses. Finally, we synthesize our findings, and recommend ways of testing our hypotheses about controls of soil microbial community stability.

## Microbial community structure, specific traits present in a community, and the R-K spectrum

Much work has been done on the relationship between the diversity and structure of microbial communities and their response to disturbance, often with contrasting results. Most evidence for relationships between microbial communities and stability (resistance or resilience under disturbance) comes from aquatic microcosm studies (e.g., Wertz et al., 2007; Wittebolle et al., 2009; Eisenhauer et al., 2012). The majority of these studies have focused on the stability of processes or bulk microbial properties (e.g., biomass or functioning) under disturbance, rather than the stability of community structure itself. Disturbance influences microbial community structure if species differ in their trade-off between growth rate and disturbance tolerance (Engelmoer and Rozen, 2009). Therefore, specific functional traits (Box 1) may be more informative of community stability in disturbed ecosystems than community composition and structure (Lennon et al., 2012; Wallenstein and Hall, 2012; Mouillot et al., 2013). For example, the ability to resist dehydration via synthesis of the sugar trehalose to maintain cell membrane integrity (e.g., McIntyre et al., 2007; Zhang and Van, 2012) may be an important soil microbial trait to consider for drought resistance, whereas the ability to use specific C or N forms that are released when a drought ends might inform about resilience (Borken and Matzner, 2009) (Table 1). In contrast, more general stress-response pathways, such as the sporulation pathway of *Bacillus subtilis* (e.g., Higgins and Dworkin, 2012) may be universally useful for maintaining stability in the face of a variety of disturbances.

**Table 1:** **Examples of microbial traits and the genes involved that might play a role in the resistance and resilience of microbial communities to climate change**.

**Trait**	**Genes involved**	**Process**	**Climate change driver**	**References**
Desiccation and heat resistance	otsBA, otsA	Trehalose synthesis Capsule	Drought, warming	Canovas et al., 2001; McIntyre et al., 2007; Miller and Ingram, 2008; Mordhorst et al., 2009; Zhang and Van, 2012
	neuO	O- acetylation		
Sporulation	>500	Multiple	Wide range of disturbances	Higgins and Dworkin, 2012
Use of specific N forms	amoA	Ammonia oxidation	Increased nitrogen availability through warming and rewetting after drought, changes in dominant N forms through warming, changes in soil moisture, and changes in soil C availability through elevated CO_2_	Lamb et al., 2011; Long et al., 2012; Yergeau et al., 2012; Yarwood et al., 2013
	cnorB	Nitric oxide reduction		
	nosZ	Nitrous oxide reduction		
	narG	Nitrate reduction		
	nirK, nirS	Nitrite reduction		
	nifH	Nitrogen fixation		
Use of specific C forms	chiA	Chitin degradation	Changes in soil C availability through rewetting after drought, and elevated CO_2_	Theuerl and Buscot, 2010; Theuerl et al., 2010; Edwards et al., 2011; Baldrian et al., 2012; Castro et al., 2012; Nannipieri et al., 2012
	mcrA	Methanogenesis		
	pmoA	Methane oxidation		
	gtlA	Citrate synthesis		
	cbhI	Cellulose degradation		
	lcc	Lignin and phenol oxidation		
	β glu	Glucose oxidation		

Dispersal mechanisms and connectivity are important for the resilience of microbial communities because the success of regional dispersal affects the maintenance of local diversity (e.g., Matthiessen et al., 2010; Lindstrom and Langenheder, 2012). Connectedness of metapopulations has been shown to be an important factor in the response of aquatic communities to disturbance (e.g., Altermatt et al., 2011; Carrara et al., 2012), but such evidence is lacking for soils. Dispersal mechanisms are likely to play an even more important role for the recovery of microbial communities in soil because of its heterogeneous nature (Ritz et al., 2004), and low moisture content can hamper dispersal of soil microbes by spatially isolating metacommunities (Treves et al., 2003). However, soil microbes can also disperse via aboveground mechanisms. For example, fungi that rely on active dispersal through airborne spores (e.g., Roper et al., 2010) may have greater resilience than bacteria that lack more active dispersal mechanisms (Kasel et al., 2008; but see Barcenas-Moreno et al., 2011). On the other hand, bacteria, archaea, and phytoplankton cells are thought to passively disperse easily because of their large populations and small body sizes (e.g., Baas-Becking, 1934; Finlay and Clarke, 1999).

From the above, we infer that specific microbial traits are pivotal for determining microbial community response to disturbance, and that the ability of a microbial community to resist or recover from a specific disturbance may be informed by the dominance, or community-weighted mean, of a specific functional trait (e.g., Wallenstein and Hall, 2012) (Table 1). Recent advances in sequence-based metagenomics allow for identification of functional genes in a microbial community (Thomas et al., 2012). However, although the presence and expression of specific functional genes in soil microbial communities has been shown to respond to global change and climate change disturbances (e.g., Baldrian et al., 2012; Yergeau et al., 2012; Yarwood et al., 2013), the relative abundance of functional genes has never been used to infer a community's ability to withstand and recover from disturbances. This approach still has many caveats; newly discovered gene sequences often lack homology to known genes in current databases and remain unknown until biochemical characterization and annotation of their functional abilities, and microorganisms may carry the genetic capacity to exhibit a certain functional trait, but, ultimately, not express the gene or produce an active gene product in nature. Thus, to capitalize on sequence-based metagenomic tools for the understanding of functional traits, the traits of interest and their genes and regulatory pathways must be well-characterized.

In addition to specific traits, microorganisms can be characterized according to their life-history strategy: r-strategists (termed ruderals in plant ecology, and copiotrophs in microbial ecology) have high growth rates and low resource use efficiency, and K-strategists (termed competitors in plant ecology, and oligotrophs in microbial ecology) have low growth rates and high resource use efficiency (Klappenbach et al., 2000; Fierer et al., 2007). This assumed fundamental trade-off between growth rate and resource use efficiency (Hall et al., 2009) may underlie the capacity of microbial communities to respond to disturbance (Schimel et al., 2007; Wallenstein and Hall, 2012), as community structure will change if the taxa present differ in this trade-off (Engelmoer and Rozen, 2009). There is evidence from both plant and soil communities that K-strategists are more resistant, but less resilient, to climate change-related disturbances than r-strategists (Grime, 2001; Haddad et al., 2008; Bapiri et al., 2010; De Vries et al., 2012a; Lennon et al., 2012), and a trade-off between resistance and resilience is widely documented (Pimm, 1984; Hedlund et al., 2004; De Vries et al., 2012a). Different soils with different microbial communities have been compared in their response to disturbances (mostly in terms of bulk biomass and function), and changes in the abundances or relative contributions of community members have been linked to the overarching stability of the microbial community structure itself (Griffiths and Philippot, 2013). As some taxa may be more sensitive to certain disturbances than other taxa, it is possible that their differential responses impact not only the abundances of insensitive community members (for instance, through changes in the strengths of microbial interactions, such as the release of an insensitive taxon from competition due to the decrease in abundance of a taxon sensitive to disturbance), but also the overarching resistance and resilience of the community. Here, we propose that community-level measures that have a theoretical relationship with a specific functional trait, or with the r-K-strategist spectrum, might predict the response of soil microbial community structure to pulse disturbances associated with climate change.

### Hypothesis 1: the resistance of microbial community structure to disturbance increases with increasing relative abundance of K strategists (or oligotrophs), but the resilience decreases.

Gram-positive bacteria often are slower growing than Gram-negative bacteria (Prescott et al., 1996), and therefore the ratio between Gram-positives and Gram-negatives of a soil microbial community might be indicative of the prevalence of K-strategists in that community. In addition, the ability of many Gram-positive bacteria to sporulate allows them to withstand a variety of disturbances, including drought (Drenovsky et al., 2010; Higgins and Dworkin, 2012). Therefore, we propose that the resistance of microbial community structure will increase with increasing Gram-positive/Gram-negative ratio, or increasing relative abundance of Gram-positive bacteria.

Similarly, microbial communities that have a high proportion of fungi compared to bacteria are associated with nutrient [N and phosphorus (P)] poor conditions that require high resource use efficiency, and fungi typically are considered to be slower growing than bacteria (Six et al., 2006). Therefore, we argue that the fungal/bacterial ratio of a soil microbial community may also be indicative of the prevalence of K-strategists in that community, and, following this, the resistance of microbial community structure will increase with increasing fungi-to-bacteria (F/B) ratio, or increasing relative abundance of fungi, whereas the resilience will decrease. The carbon-to-nitrogen (C/N) ratio of microbial communities may be also be linked to intrinsic growth rate; fungi are slower-growing and have wider C/N ratios than bacteria (Van Veen and Paul, 1979; Bloem et al., 1997; but see Cleveland and Liptzin, 2007), thus, microbial communities that are dominated by fungi rather than bacteria will have a wider C/N ratio.

Finally, the resilience of microbial community structure will increase with increasing abundance of bacteria that can be classified as copiotrophs, such as many members of the β-proteobacteria and Bacteriodetes, and decreasing abundance of oligotrophs, such as many members of the Acidobacteria (Fierer et al., 2007). Notably, many oligotrophic microorganisms may be r-strategists, while many copiotrophic microorganisms may also be K-strategists, and so there is likely overlap between the two types of classification. Although we propose here that the above community attributes can be used to predict the resistance and resilience of microbial community composition, we acknowledge that within the categories and distinctions we propose, there will of course be exceptions that do not respond as we suggest.

At first, it may seem circular that quickly-growing organisms will be less resistant but more resilient to disturbances, and that communities with frequent disturbance regimes may be dominated by microorganisms exhibiting these strategies because of selection. However, we believe that our hypothesis is not merely self-affirming because microorganisms may respond to disturbances not only by growing and dying, but also, for example, by temporarily changing their physiological state or metabolism (e.g., entering dormancy), maintaining stochastic gene expression, exhibiting phenotypic plasticity, or being rescued by dispersal from nearby meta-communities (e.g., Shade et al., 2012a). Therefore, given the array of complex responses that microorganisms may have when challenged with a disturbance, growth is not the only mechanism that could maintain community stability.

## Higher trophic levels

Although there is some evidence from aquatic and terrestrial studies that the presence of higher trophic levels can enhance the recovery of microbial biomass and activity (Maraun et al., 1998; Downing and Leibold, 2010), almost no attention has been given to the role of higher trophic levels of the soil food web in controlling resilience of microbial community structure. Microbial grazers have the potential to affect resilience of microbial community structure via two mechanisms. First, they can aid the dispersal of microbes by carrying them in their guts or on their surfaces. For example, bacterial-feeding nematodes disperse bacteria by carrying them both their surfaces and in their guts (Ingham, 1999), fungal spores are dispersed by the movement of fungal grazers such as collembolans (Renker et al., 2005), and bacterioplankton may “hitchhike” on zooplankton carapaces to overcome otherwise impenetrable gradients in water columns (Grossart et al., 2010). In addition, microbial grazers affect microbial communities by preferentially feeding on specific taxa or functional groups, thereby either reducing their abundance or stimulating their turnover and activity (Chen and Ferris, 2000; Cole et al., 2004; Fu et al., 2005; Postma-Blaauw et al., 2005). As an example, heterotrophic nanoflagellates, prominent bacteriovores in aquatic systems, often preferentially graze on medium-sized bacterioplankton, leaving the small and large-bodied organisms behind (Miki and Jacquet, 2008).

### Hypothesis 2: the resilience of microbial community structure increases with greater diversity of organisms of higher trophic levels

Different microbial grazers have different feeding preferences, and different soil faunal species often have different movement patterns. Thus, we hypothesize that a greater diversity or species richness of higher trophic levels in the soil food web enhances resilience of soil microbial communities after disturbance, because they stimulate the growth and dispersal of a wider range of soil microbes than faunal communities of lower diversity.

## Resource availability

As suggested by Wallenstein and Hall (2012) resource availability might constrain the rate of soil microbial community adaptation and recovery; in low resource environments, shifts in microbial community structure will be slow, whereas in high resource environments, communities will respond rapidly. Indeed, resource availability has been linked to resilience of microbial and faunal biomass several times (Orwin et al., 2006; De Vries et al., 2012b). It was observed (but not quantified in regards to community composition) that the resilience of both microbial and faunal communities seemed to be increased by the presence of plants (De Vries et al., 2012b) presumably because plants offer substantial belowground carbon inputs for microbial communities. Resource availability has the potential to both enhance and retard microbial community resilience, depending on the remaining microbial traits after a disturbance: low resource availability may give slow-growing (oligotrophic) microbes a competitive advantage, whereas high resource availability may favor fast-growing (copiotrophic) microbes. Therefore, we propose that a greater resource availability, diversity, and heterogeneity would increase community resilience after a disturbance, and indeed, several studies report a positive effect of plant species diversity (with presumably a diversity of belowground root exudates and litter inputs) on the stability of microbial biomass and microbial processes (Milcu et al., 2010; Royer-Tardif et al., 2010). Moreover, root exudates form a tight evolutionary link between plants and microbial communities (Badri and Vivanco, 2009), and recent evidence showed that different chemical compositions of *Arabidopsis* root exudates select for different microbial communities (Badri et al., 2013), thereby potentially affecting the response of those communities to climate change. Because plants respond to climate change by modifying their C balance (Atkin and Tjoelker, 2003; Chaves et al., 2003), temporal changes in root exudation especially have great potential to affect microbial community responses to climate change.

### Hypothesis 3: the resilience of microbial community structure increases with greater resource availability. because of the belowground C inputs by plant, the presence of a plant will increase the resilience of the microbial community

Increased concentrations of labile carbon, nitrogen, and phosphorus as a result of greater resource availability might allow microbial taxa to maximize their intrinsic growth rate and thus increases the resilience of microbial community composition. We also hypothesize that the presence of a plant enhances the resilience of microbial community structure through its belowground carbon inputs.

## Moisture availability

Moisture availability plays a crucial role for microbial activity and survival, because microbes are in close contact with water and have semi-permeable cell walls. In addition and as briefly mentioned earlier, low soil moisture content limits the dispersal of microorganisms (Carson et al., 2010; Kravchenko et al., 2013). However, moisture is also limiting for the movement of microbial grazers such as nematodes (Young et al., 1998), which, as hypothesized above, might promote growth and dispersal of microbes and increase microbial community resilience.

### Hypothesis 4: moisture availability increases resilience of microbial community structure

We hypothesize that relatively higher moisture availability increases the recovery of microbial community structure after drought, and also after other types of disturbance, such as changes in N and C availability (as a result of increased atmospheric CO_2_ concentrations) or heat waves.

## Methods

We analyzed three case studies to test the hypotheses about soil microbial community resistance and resilience outlined above, focusing on drought, rainfall, and increased litter inputs. In all three case studies, we calculated Bray-Curtis similarities between disturbed and control microbial communities as a measure of both resistance and resilience of microbial community structure. For resistance, this was the similarity between the disturbed treatment and the control at the end of the disturbance; for resilience, it was the similarity between the disturbed treatment and the control after ending the disturbance. In both cases, a similarity of 1 would mean maximum resistance (no effect of disturbance) or resilience (complete recovery). We used axis scores from ordination plots as metrics of microbial community structure, as well as F/B ratio and Gram-positive/Gram-negative ratio. We fitted single-variable linear and non-linear models [including a quadratic term of the significant explanatory variable(s)] (lm function in R) to explain resistance and resilience from metrics of microbial community structure, as well as from higher trophic level richness and numbers, soil C and N availability, and soil moisture content. If the quadratic term was significant, we performed an ANOVA to test whether the non-linear model significantly improved model fit. Finally, we fitted the best explaining additive model for microbial community resistance and resilience using parameters that had shown to be significant in the single-variable models. All analyses were performed in R [version 2.15.2, (2012)].

## Case study 1: responses of grassland and wheat field microbial communities to multiple drought events

The data from case study 1 were originally published in two papers: De Vries et al. (2012a) and De Vries et al. (2012b). The experiment investigated the responses of the entire soil food web and of C and N cycling in grassland and wheat soil to drought. The experiment included two phases: a field-based drought and a glasshouse-based drought. During the glasshouse-based experiment, the response of biomass of functional groups and processes in both control and drought treatments was monitored directly 1, 3, 10, and 77 days after ending the drought. This, in combination with 32 experimental units (land use × field drought × glasshouse drought × 4 replicates) per sampling, and an extra set of pots in which a wheat plant was grown to assess the impact of plant presence on the recovery of the soil food web, resulted in a total of 192 observations. Microbial communities were analyzed using analysis of phospholipid-derived fatty acid profiles (PLFA). In addition, soil concentrations of available C, N, and moisture were measured, as well as leaching and gaseous losses of C and N. For more details on methods and experimental set up see De Vries et al. (2012a,b).

The original publications focused on the impact of drought on biomass and activity of soil food webs, with only a minor role for changes in community composition. The biomass and activity of fungal-based soil food webs of grasslands were found to be more resistant to drought, whereas biomass and activity bacterial-based soil food webs were more resilient. In addition, the presence of a plant increased the resilience of microbial biomass, and resilience of microbial biomass was positively related to C availability. Here, we re-analyzed microbial community data to test our four hypotheses about resistance and resilience of microbial community structure. We calculated F/B ratio (the ratio between the fungal PLFA 18:2ω 6 and the bacterial PLFAs i-15:0, a-15:0, 15:0, i-16:0, 16:1ω 7, 17:0, a-17:0, cyclo-17:0, 18:1ω 7, and cyclo-19:0), Gram-positive/Gram-negative ratio (the ratio between Gram-positive PLFAs i-15:0 and i-17:0 and Gram-negative PLFAs a-C15:0, 16:1ω 7, cyclo-17:0, and cyclo-19:0) and PCA scores of relative abundances of PLFAs [widely used in ecology for analyzing PLFA profiles, e.g., in De Vries et al. (2012c)].

We found that both the resistance and the resilience of microbial communities were explained by community structure. In line with hypothesis 1, resistance decreased with greater PC1 scores, along which Gram-negative abundance increased (Table A4), and increased with greater Gram positive/Gram negative ratio (quadratic relationship, Table 2). However, resistance decreased with greater F/B ratio, which is in contrast with hypothesis 1, and with earlier findings that resistance of biomass and activity to drought increased with greater relative abundance of fungi (Bapiri et al., 2010; De Vries et al., 2012a). A possible explanation for this is that there is only one PLFA that represents fungi, whereas there are ten PLFAs for bacteria. Thus, changes in microbial community structure therefore are dominated by changes in the bacterial members, and the ratio between fungal and bacterial PLFA might not be the most informative for those changes. In addition, the bacterial community in a fungal-dominated microbial community might undergo more dramatic shifts in composition because of intense competition with fungi.

**Table 2:** **Case study 1: regression models explaining microbial community resistance to the glasshouse-based drought**.

**Model**	**Intercept**	***P***	**Independent variables included in model**	**Parameter value**	***P***	**Adj. *R*^2^**
Single, linear	0.93	<0.0001	PC1 scores	−0.008	<0.0001	0.79
Single, linear	1.01	<0.0001	F/B ratio	−1.48	0.0005	0.56
Single, non-linear	0.75	<0.0001	Gram+/gram− ratio	+3.7 ^*^ 10^−3^	<0.0001	0.88
			(Gram+/gram− ratio)^2^	−1.8 ^*^ 10^−5^	<0.0001	
Multiple, non-linear	0.83	<0.0001	PC1	−5.0 ^*^ 10^−3^	0.034	0.91
			Gram+/gram− ratio	+2.3 ^*^ 10^−4^	0.006	
			(Gram+/gram− ratio)^2^	−1.3 ^*^ 10^−5^	0.002	

In contrast to hypothesis 1, resilience decreased with greater PC1 scores, whereas it increased with greater C/N ratio of microbial biomass and greater F/B ratio (included in best model, Table 3) and Gram positive/Gram negative ratio (Table 3). The positive relationship between resilience and F/B ratio as well as Gram-positive/Gram-negative ratio might reflect the fact that the initial changes in communities dominated by fungi and Gram-positives were smaller and thus these remained more similar to their undisturbed counterparts throughout. This is further supported by the lack of evidence for a trade-off between resistance and resilience. In comparison, the resilience index proposed by Orwin et al. (2010) calculates the resilience relative to the initial change in a parameter, and thus a low resistance is more likely followed by a high resilience. It goes beyond the scope of this paper to compare the use of different resilience indices, but it is noteworthy that different methods of calculating these indices can give different results.

**Table 3:** **Case study 1: regression models explaining variation in microbial community resilience after the glasshouse-based drought**.

**Model**	**Intercept**	***P***	**Independent variables included in model**	**Parameter value**	***P***	**Adj. *R*^2^**
Single, linear	0.93	<0.0001	Microarthropod richness	+0.004	0.001	0.12
Single, linear	0.96	<0.0001	Protozoa numbers	−7.0 ^*^ 10^−8^	<0.0001	0.33
Single, linear	0.95	<0.0001	PC1	−0.005	<0.0001	0.50
Single, linear	0.93	<0.0001	Microbial biomass C/N	+0.006	0.009	0.07
Single, non-linear	0.91	<0.0001	Gram+/gram− ratio	+4.1 ^*^ 10^−4^	0.006	0.16
			(Gram+/gram− ratio)^2^	−6.2 ^*^ 10^−6^	0.05	
Multiple, linear	0.91	<0.0001	Protozoa numbers	−5.1 ^*^ 10^−9^	<0.0001	0.63
			PC1	−4.1 ^*^ 10^−3^	<0.0001	
			Gram+/gram− ratio	+3.2 ^*^ 10^−4^	0.001	
			F/B ratio	0.34	0.002	

**Figure 1:**
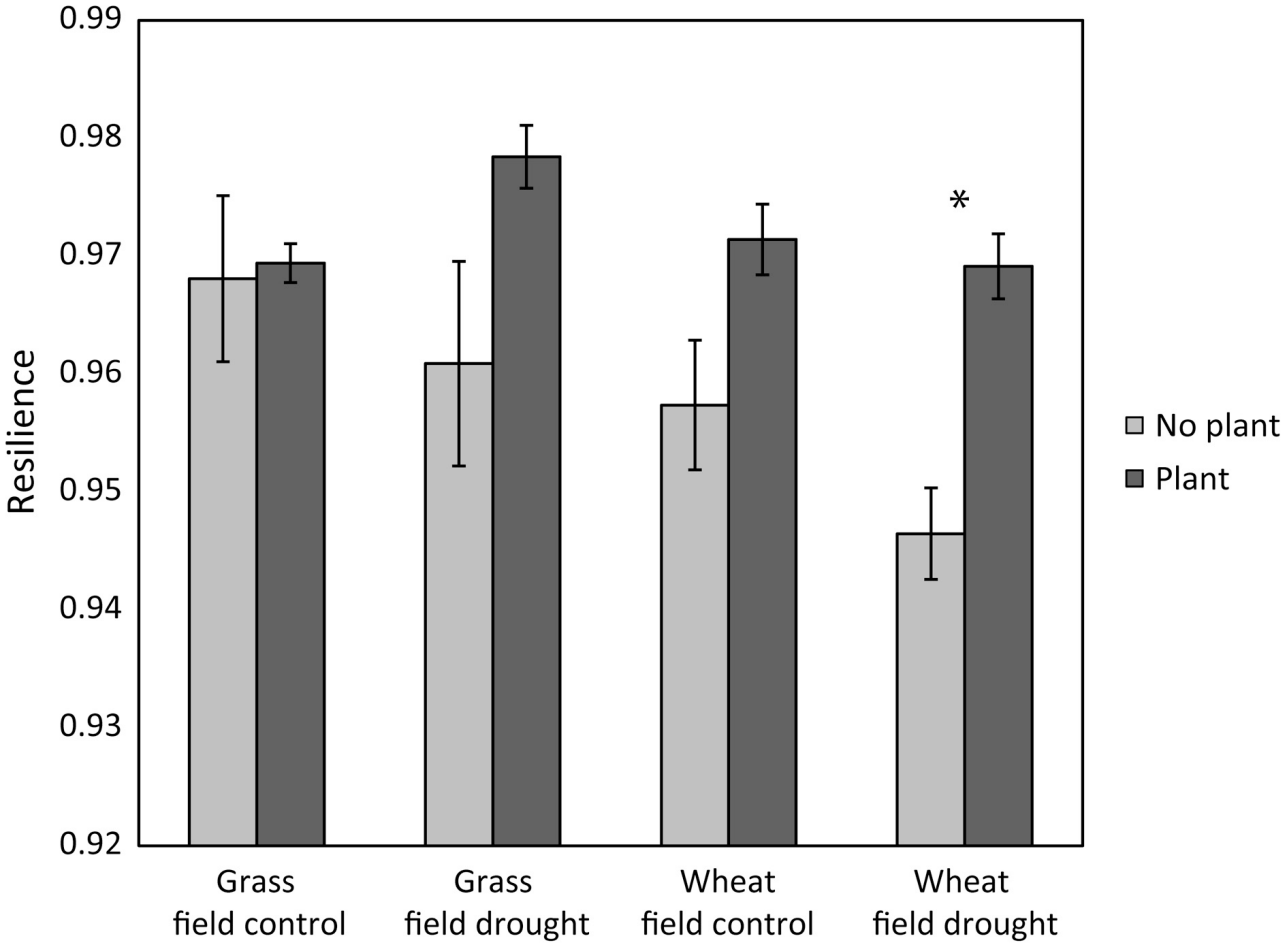
**Case study 1: the presence of a plant increased the resilience of microbial community composition 77 days after ending the glasshouse-based drought [*F*_(1, 24)_ = 15.7, *P* = 0.0005].** Resilience was greater in grassland than in wheat [*F*_(1, 24)_ = 5.36, *P* = 0.029]; there were no interaction effects between land use or previous drought. Pairwise comparisons within land use and field drought treatments indicated that only within the wheat field drought treatment the treatments with and without plant were (marginally) significantly different (Tukey's HSD comparison, *P* = 0.059, indicated by an asterisk).

Our results partly support hypotheses 2, 3, and 4. As hypothesized, resilience of microbial community structure increased with greater microarthropod richness. However, it decreased with greater protozoa numbers (Table 3). When only the last sampling (77 days after ending the drought) was analyzed, the positive relationship of resilience with greater microarthropod richness was also significant (adjusted R-squared = 0.30, *P* = 0.017), but resilience increased with protozoa numbers (adjusted R-squared = 0.22, *P* = 0.037). Notably, the presence of a plant strongly increased overall microbial community resilience, although within land use and field drought treatments this effect was not, or only marginally, significant (Figure 1). Within the plant treatment, resilience increased with increasing soil dissolved organic C availability (adjusted R-squared = 0.22, *P* = 0.038). These results support our hypothesis that plant belowground C inputs increase microbial community resilience. However, the lack of explanatory power of overall resource availability for community resilience might indicate that other mechanisms are more important, such as the greater abundance of higher trophic levels in plant treatments (De Vries et al., 2012b), or plant impacts on soil structure and aeration, which were not measured here.

## Case study 2: response of microbial communities from intensively managed and extensively managed grassland to drought

In the study published by Gordon et al. (2008) the impact of a glasshouse-based drought was assessed on microbial communities from extensively managed, unfertilized, species rich grassland, and from intensively managed, fertilized, and heavily grazed grassland, alongside measurements of C and N leaching. The response of microbial biomass C and N, and C and N leaching, was measured 1, 3, 9, 16, 30, and 50 days after rewetting, while microbial community structure (as PLFAs) was measured only at day 30. With two land uses, a drought vs. a control, and four replicates, this resulted in 16 observations for microbial community structure.

In the original publication, the authors found that biomass N of the (fungal-dominated) microbial community of extensively managed grassland was less affected by drought than that of the bacterial-dominated microbial community of intensively managed grassland. Moreover, this was paralleled by smaller leaching losses of C and N from the grassland soil. Changes in microbial community composition were not analyzed quantitatively. Here, we re-analyzed microbial community data to test our hypotheses that microbial community resilience can be explained by microbial community structure. As in case study 1, we used PCA scores as microbial community metrics, alongside F/B ratio and Gram-positive/Gram-negative ratio.

The results from this case study support hypothesis 1. We found that resilience was negatively related to the F/B ratio and the Gram-positive/Gram-negative ratio. In addition, resilience increased with greater PC1 scores (Table 4), along which most Gram-negative PLFAs increased and fungal PLFA decreased (Table A5). This dataset did not allow for testing the other hypotheses.

**Table 4:** **Case study 2: regression models explaining variation in microbial community resilience at day 30 after ending the glasshouse-based drought**.

**Model**	**Intercept**	***P***	**Independent variables included in model**	**Parameter value**	***P***	**Adj. *R*^2^**
Single, linear	0.95	<0.0001	F/B ratio	−3.76	0.0094	0.65
Single, linear	0.94	<0.0001	PC1 scores	+0.003	0.013	0.62
Single, non-linear	1.04	<0.0001	Gram+/gram− ratio	−0.137	0.021	0.77
			(Gram+/gram− ratio)^2^	+0.0359	0.028	
Single, linear	0.96	<0.001	Microbial biomass	−1.7 ^*^ 10^−5^	0.024	0.53

## Case study 3: tropical forest soil microbial communities responses to litter addition, litter removal, and rainfall exclusion in a field experiment

Nemergut et al. (2010) published a study assessing the impact of organic matter content through on soil microbial communities in Costa Rican tropical forest soils. The design included three experimental treatments (litter exclusion, litter addition, and throughfall exclusion) and one control, each observed over time in triplicate plots. The control plots were sampled at the beginning of the experiment, in April 2007, and then subsequently in June and October 2008. The experimental plots were sampled in June and October 2008, resulting in 27 total observations. Pyrosequencing of the 16S rRNA gene was used to measure of bacterial and archaeal community structure, and a suite of soil environmental parameters were also assessed, including: soil water content, microbial biomass, CO_2_ efflux, dissolved oxygen, and ammonium and nitrate concentrations. The sequencing data, contextual data, and metadata were deposited in MG-RAST and made publicly available. The Nemergut et al. (2010) dataset was selected as a case study because parameters of interest to global change disturbance were measured (microbial community structure, soil resources, and soil moisture), and because it provided a sequence-based assessment of composition to complement the PLFA-based assessments of Case Studies 1 and 2.

In the original work, the authors reported that certain phyla of bacteria and archaea were more prevalent in some of the experimental treatments than others, and, more specifically, that oligotrophic taxa (e.g., Acidobacteria) were more prevalent in plots that were compromised in organic matter availability. To query the dataset specifically about community resistance and resilience, we first calculated resistance as the Bray-Curtis similarity (averaged across replicates) between the initial time point (pre-disturbance) control and the post-manipulation time point for each experimental treatment (April control vs. June treatment). Then, we calculated resilience as the Bray-Curtis similarity between the final time point and the April pre-disturbance control (April control vs. October treatment). We used unconstrained correspondence analysis to determine axis scores as a metric of microbial community structure.

We found that microbial community structure (axis 1 CA scores) explained variability in resistance across treatments (non-linear model: resistance was explained by main and quadratic term of axis 1 scores, adjusted R squared = 0.89, *p* < 0.0001 and *p* = 0.004, respectively)—resistance increased with axis 1 scores. The axis 1 gradient corresponded to transition from communities with a high representation of Proteobacteria-affiliated taxa (many of which can be classified as copiotrophs) to communities with a high representation of Acidobacteria-affiliated taxa (many of which can be classified as oligotrophs; Table A6 online Suppl. Data). Thus, this result supports hypothesis 1 that resistance increases with increasing abundance of oligotrophs. Axis 2 CA scores and microbial biomass did not provide explanatory value for resistance. Of all the available environmental measurements, only nitrate concentrations and moisture content explained variability in resilience (Pearson's correlation between moisture and nitrate −0.123, *P* = 0.538); resilience increased with nitrate availability, but decreased with moisture content (Table 5). This suggests that nitrate availability and moisture are important for resilience of microbial communities in tropical soils, and supports hypothesis 3, but not hypothesis 4, which pose that resilience increases with nutrient and water availability, respectively. The dataset did not allow for testing the remaining hypotheses.

**Table 5:** **Case study 3: regression models explaining variation in microbial community resilience after litter addition, litter removal, and rainfall exclusion**.

**Model**	**Intercept**	***P***	**Independent variables included in model**	**Parameter value**	***P***	**Adj. *R*^2^**
Single, linear	0.22	<0.0001	Nitrate	+0.006	0.026	0.34
Single, non-linear	0.44	<0.0001	Moisture	−0.004	0.005	0.52
Multiple, linear	0.40	<0.0001	Nitrate	+0.004	0.023	0.71
			Moisture	−0.035	0.005	

Notably, there were only small changes in community composition within treatments over time, which prompted the authors to combine the time points for their original analysis. This is, in some ways, expected because spatial variability often exceeds temporal variability in soil communities (Bardgett et al., 1997; Ettema and Wardle, 2002). However, the resistance and resilience determined by these small changes were well-explained by community structure, nitrate, and water content.

## Support for the hypotheses – a framework for predicting microbial community resistance and resilience to climate change

**Figure 2:**
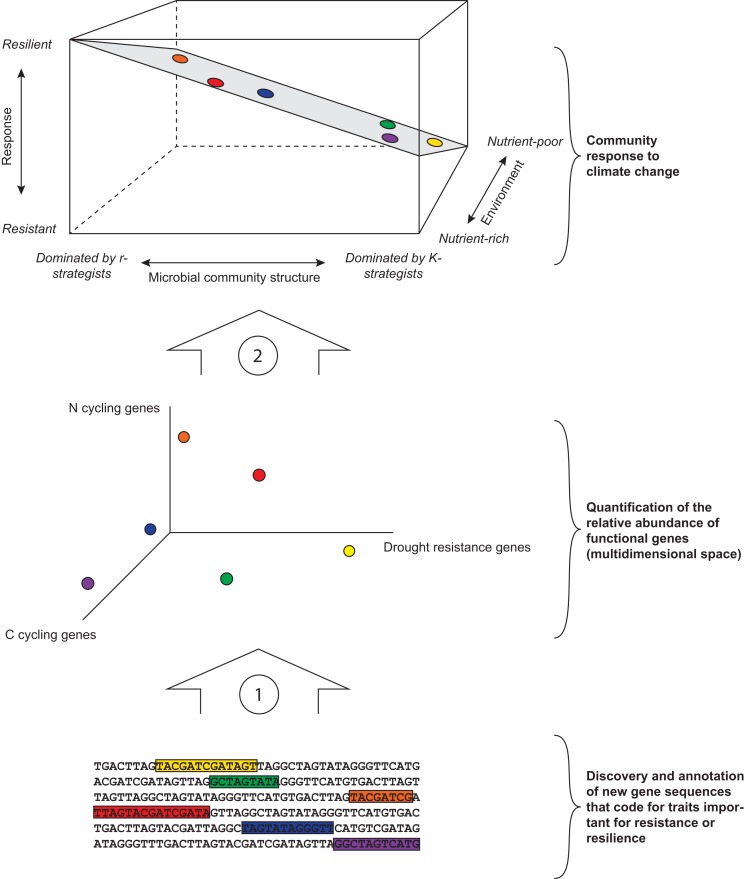
**Framework for predicting microbial community response to climate change.** The bottom part of the figure illustrates the necessity of characterizing and annotating specific functional genes (here conceptually represented by colored sequences) that code for microbial traits of importance for community responses for specific disturbances associated with climate change. Once known and annotated, these genes can inform about the relative abundance of a suite of genes that may underlie a community's response to climate change (arrow 1). The middle part designates the relative abundance of functional genes present in a community. This space is multidimensional and here we chose to visualize C cycling genes, N cycling genes, and drought resistance genes (see Table 1), but other known and unknown genes such as those involved in sporulation or specific dispersal mechanisms should be included. The functional genes present in a community may, or may not, have a relationship with the dominance of r- and K-strategists or with the community's environment (colored dots in middle and upper part). The role of specific functional genes in a community's response and their links with the r-K spectrum are yet to be elucidated (arrow 2). The upper part of the figure indicates a community's response to climate change, as determined by the relative abundance of r- and K-strategists and the community's environment (in this case nutrient availability, but this can be replaced by other environmental factors such as the abundance or richness of higher trophic levels). A K-strategist dominated microbial community in a nutrient-poor environment likely has high resistance, whereas an r-dominated community in a nutrient-rich environment likely has high resilience. The exact shape of the surface might vary depending on specific circumstances.

In all three case studies, the resistance and the resilience of microbial communities could be explained by community properties associated with the r-K spectrum. We found that the measures that significantly explained resistance and resilience and were indicative for shifts from r-strategists to K-strategists were strongly interrelated (Tables A1–A3), confirming that these measures inform about broad shifts in community structure linked to changes in the abundance of r- and K-strategists. Moreover, the presence and abundance of higher tropic levels, resource availability, and moisture content were strong predictors for microbial community resilience. Although the structure of the data we analyzed does not allow for drawing conclusions on the relative importance of those controls, and the relationships we found are not necessarily causal, these results are a first observation and exploration of a framework for predicting the response of soil microbial communities to climate change based on the ratio between r- and K-strategists, and the environment (Figure 2, top panel). We propose that, although the underlying specific functional genes present in a microbial community determine its response to climate change, simple measures that characterize microbial communities along the r-K spectrum can inform its ability to resist and recover from climate change related disturbances. Our framework also takes into account the effect of the environment, and interrelationships between environment and r-K dominance of microbial communities, in the three-dimensional response plane.

Furthermore, we propose that the abundance of specific functional genes such as those involved in desiccation resistance will predict a community's response to drought, but genes involved in C and N cycling might link to the r-K spectrum and thus be useful for predicting microbial community response to climate change (Table 1; Figure 2). For example, the abundance of amoA genes is likely to be greater in N-poor environments in which the dominant N form is ammonia than in nutrient rich environments in which the dominant form is nitrate (Schimel and Bennett, 2004), and might thus be associated with microbial communities dominated by oligotrophs. Ultimately, our framework allows for plotting specific functional traits onto this plane for predicting microbial community stability under a range of specific disturbances.

## Future directions: the roles of multiple disturbances and adaptation for soil microbial community stability

By selecting for specific traits among community members, a disturbance may affect a community's ability to respond to a subsequent disturbance or to a series of compounded disturbances. For example, it has been shown that the order of different types of disturbances influences the outcome of community structure, suggesting that selection for a specific trait affects the ability to respond to a subsequent disturbance of a different type (Fukami, 2001). Thus, we may expect that when a microbial community is exposed to two subsequent disturbances of the same type, its composition will be more resistant to the second disturbance because of selection for the tolerant trait by the first disturbance. There is some support for this hypothesis from soils. Precipitation regime affected the response of soil bacterial community composition to subsequent drought and rewetting events (Evans and Wallenstein, 2012), and extremophiles are often tolerant to a wide range of disturbances (Mangold et al., 2013). In contrast, microbial communities exposed to severe drought appeared to be more resistant to a subsequent heat wave, suggesting that the microbial traits responsible for drought tolerance are related to those of heat-tolerance (Berard et al., 2012). However, very little is known about the interrelatedness between specific functional traits in soil microbes, which makes it difficult to predict responses to multiple disturbances. In contrast, the r-K spectrum might inform about a microbial community's ability to withstand different types of disturbance: r-strategists thrive in nutrient (N and P) rich, disturbed environments compared to K-strategists, but are less resistant to climate change than K-strategists (Hedlund et al., 2004; De Vries et al., 2012a).

Adaptation also may be an important strategy for individual microbial taxa to cope with a changing climate (Box 1). A microbe's ability to adapt to disturbance is linked to its generation time or turnover rate, and therefore r-strategists may show quicker adaptation than K-strategists. Moreover, warming can increase growth rates, but also horizontal gene transfer between bacterial taxa (Pritchard, 2011). In addition, for example, it has been shown that *E. coli* can acquire stress resistance to a range of disturbances after pretreatment with a different disturbance after only 500 generation times (Dragosits et al., 2013). This so called cross-stress protection has been shown for a range of species across kingdoms. Similar to microbial community resilience, rates of adaptation and evolution are likely influenced by environmental factors such as the abundance and richness of higher trophic levels, moisture availability, and resource availability. Although not within the scope of this paper, these findings suggest that evolutionary changes might be of equal importance to shifts in community structure for determining the response of microbial communities to climate change (Orsini et al., 2013).

## Conclusion

Our aim in this paper was to hypothesize controls on microbial community resistance and resilience to climate change, and to explore our hypotheses by carefully re-analyzing three vignettes of published data. Our results show that both microbial community properties associated with the r-K spectrum and environmental factors such as the abundance and richness of higher trophic levels, plant presence, and resource availability can explain the response of microbial community structure to climate change-related disturbances. A clear limitation to our study is the relatively narrow focus on three vignettes of case studies, and further investigation and experimental validation is necessary to directly test these hypotheses across a wide range of soil ecosystems. Although querying publicly available data can be used to formulate hypotheses on the potential controls of microbial community resistance and resilience, disentangling the interwoven controls on microbial community resistance and resilience requires mechanistic experiments designed to test specific questions about the hypothesized controls (Jansson and Prosser, 2013).

As a final consideration, it is possible that routine successional trajectories of microbial communities (for example, seasonal trajectories in temperate soils) may be altered permanently as a result of a disturbance. However, the nature of these alterations will depend on the traits present in the community and on the type of disturbance. In temperate aquatic systems, it has been suggested that annual seasonal succession in bacterial community composition may serve as a baseline from which a community's response to a pulse disturbances can be measured, while gradual shifts in this succession may be used as an indicator of long-term adaptations to press disturbances such as global climate changes (Shade et al., 2012a,b). Similarly, soil community successional trajectories may be quantified and monitored to detect gradual shifts in composition over the long term, such as in response to the press disturbance of increased temperature, and how these shifts affect short-term responses to pulse disturbances, such as drought. However, typical rates of community turnover in soil systems are not well documented, especially at the same site on inter-annual scales, and in the absence of any disturbance (Shade et al., 2013). Knowledge of these baseline seasonal dynamics for soils is crucial for providing context for community responses to pulse disturbances, like drought and flooding. Therefore, collecting time series of soil communities and quantifying baseline fluctuations should be prioritized toward the goal of further understanding microbial community stability given ongoing and compounded global climate change disturbances. Combined with long-term experiments that directly manipulate anticipated global change disturbances [e.g., free-air carbon dioxide enrichment experiments (Ainsworth and Long, 2005)], we think that these time series will provide essential insights into the important microbial traits and environmental conditions that may alter or maintain ecosystem services in the face of global changes.

### Conflict of interest statement

The authors declare that the research was conducted in the absence of any commercial or financial relationships that could be construed as a potential conflict of interest.

## Figures and Tables

**Table A4:**
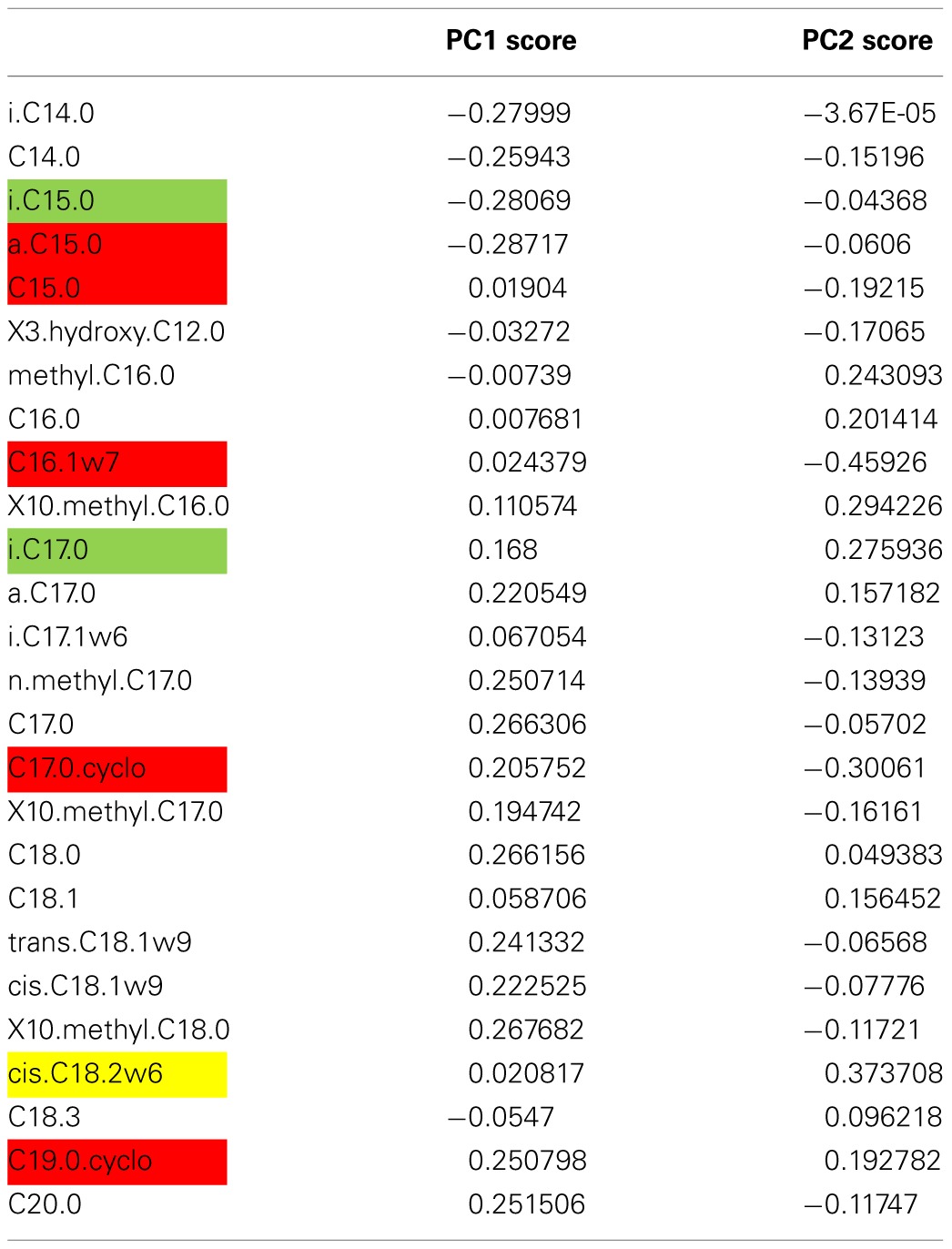
**Axis loadings of individual PLFA in Case study 1**.

Axis 1 explained 43% of variation, axis 2 explained 14% of variation. PLFAs marked green, red, and yellow are representative of Gram-positive, Gram-negative, and fungi, respectively.

**Table A5:**
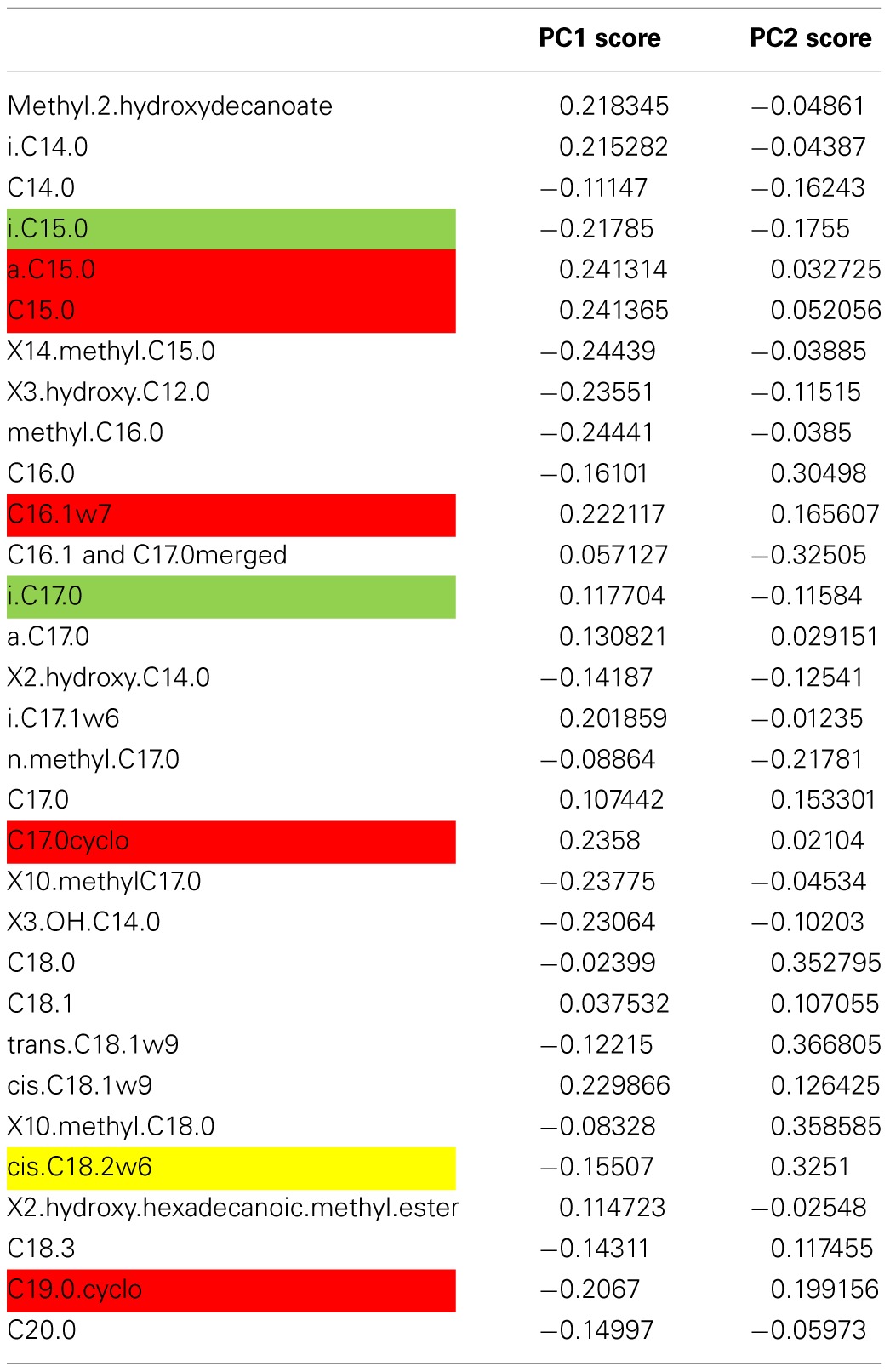
**Axis loadings of individual PLFA in Case study 2**.

Axis 1 explained 53% of variation, axis 2 explained 15% of variation. PLFAs marked green, red, and yellow are representative of Gram-positive, Gram-negative, and fungi, respectively.

## Appendix

**Table A1:** **Pearson correlation coefficients between variables explaining microbial community resistance in Case study 1**.

	**PC1 axis**	**F/B ratio**	**Gram+/Gram− ratio**
PC1 axis			
F/B ratio	0.75		
Gram+/Gram− ratio	−0.87	1.7 ^*^ 10^−5^	

Underlines values designate significant correlations (P < 0.05).

**Table A2:** **Pearson correlation coefficients between variables explaining microbial community resilience in Case study 1**.

	**Protozoa**	**Micro arthropods**	**PC1**	**F/B ratio**	**Gram+/Gram− ratio**	**Microbial C/N ratio**
Protozoa						
Microarthropods	−0.35					
PC1	0.43	−0.35				
F/B ratio	0.26	0.10	0.36			
Gram+/Gram− ratio	−0.08	0.015	−0.43	−0.67		
Microbial C/N ratio	−0.09	0.18	−0.25	−0.21	0.21	

Underlines values designate significant corrections (p < 0.05).

**Table A3:** **Pearson correlation coefficients between variables explaining microbial community resilience in Case study 2**.

	**F/B ratio**	**PC1**	**Gram+/Gram− ratio**	**Microbial biomass**
F/B ratio				
PC1	−0.71			
Gram+/Gram− ratio	0.59	−0.97		
Microbial biomass	0.63	−0.90	0.92	

Underlines values designate significant correlations (P < 0.05).

**Table A6:** **CA axis scores for the 20 most abundant bacterial taxa in Case study 3**.

**OTU_ID**	**CA1**	**CA2**	**Total no. seqs**	**Consensus lineage**
7721	−0.052755783	0.128596257	1232	k__Bacteria; p__Proteobacteria; c__Alphaproteobacteria; o__Rhizobiales; f__Hyphomicrobiaceae; g__Rhodoplanes; s__
7592	−0.277985389	0.384482383	559	k__Bacteria; p__Proteobacteria; c__Alphaproteobacteria; o__Rhizobiales; f__Bradyrhizobiaceae
5179	−0.031267675	0.274724392	477	k__Bacteria; p__Proteobacteria; c__Alphaproteobacteria; o__Rhizobiales; f__Hyphomicrobiaceae; g__Rhodoplanes; s__
4450	−0.368835276	−0.01104972	434	k__Bacteria; p__Proteobacteria; c__Deltaproteobacteria; o__Syntrophobacterales; f__Syntrophobacteraceae; g__; s__
3664	−0.203535915	0.204631553	397	k__Bacteria; p__Proteobacteria; c__Alphaproteobacteria; o__Rhizobiales; f__Hyphomicrobiaceae; g__Rhodoplanes; s__
232	0.317417102	−0.327066878	291	k__Bacteria; p__Acidobacteria; c__Acidobacteria-5; o__; f__; g__; s__
6514	0.374990616	0.045919714	275	k__Bacteria; p__Acidobacteria; c__Acidobacteria-2; o__; f__; g__; s__
3615	0.148161131	0.186057802	271	k__Bacteria; p__Acidobacteria; c__Acidobacteria-2; o__; f__; g__; s__
6194	0.272492541	−0.440098295	243	k__Bacteria; p__Nitrospirae; c__Nitrospira; o__Nitrospirales; f__Nitrospiraceae; g__Nitrospira; s__
1968	0.406077387	−0.153510689	236	k__Bacteria; p__Proteobacteria; c__Alphaproteobacteria; o__Rhizobiales; f__Hyphomicrobiaceae; g__Rhodoplanes; s__
2877	−0.452435542	0.331937119	214	k__Bacteria; p__Bacteroidetes; c__Flavobacteriia; o__Flavobacteriales; f__Flavobacteriaceae; g__Flavobacterium
5980	1.037736522	−0.623269339	211	k__Bacteria; p__Acidobacteria; c__Acidobacteria-2; o__; f__; g__; s__
3158	0.511248597	−0.360815747	177	k__Bacteria; p__Proteobacteria; c__Betaproteobacteria; o__; f__; g__; s__
741	0.561319057	−0.242946783	166	k__Bacteria; p__Acidobacteria; c__Acidobacteria; o__Acidobacteriales; f__Koribacteraceae; g__; s__
9410	0.330214073	0.053950661	162	k__Bacteria; p__Acidobacteria; c__Acidobacteria; o__Acidobacteriales; f__Koribacteraceae; g__Candidatus Koribacter; s__
4283	−0.21498302	0.197813008	158	k__Bacteria; p__Proteobacteria; c__Alphaproteobacteria; o__Rhizobiales; f__Rhodobiaceae; g__; s__
2587	−0.012292612	0.229939177	157	k__Bacteria; p__Proteobacteria; c__Alphaproteobacteria; o__Rhizobiales; f__Hyphomicrobiaceae; g__Rhodoplanes; s__
2250	0.126551331	−0.137573106	154	k__Bacteria; p__Proteobacteria; c__Deltaproteobacteria; o__Syntrophobacterales; f__Syntrophobacteraceae; g__; s__
8618	−0.03958676	0.222012965	153	k__Bacteria; p__Proteobacteria; c__Gammaproteobacteria; o__Xanthomonadales; f__Sinobacteraceae; g__; s__

CA axis 1 explained 5.47% and CA axis 2 explained 5.05% of variation.
